# Association of serum levels of antibodies against MMP1, CBX1, and CBX5 with transient ischemic attack and cerebral infarction

**DOI:** 10.18632/oncotarget.23789

**Published:** 2017-12-31

**Authors:** Hao Wang, Xiao-Meng Zhang, Go Tomiyoshi, Rika Nakamura, Natsuko Shinmen, Hideyuki Kuroda, Risa Kimura, Seiichiro Mine, Ikuo Kamitsukasa, Takeshi Wada, Akiyo Aotsuka, Yoichi Yoshida, Eiichi Kobayashi, Tomoo Matsutani, Yasuo Iwadate, Kazuo Sugimoto, Masahiro Mori, Akiyuki Uzawa, Mayumi Muto, Satoshi Kuwabara, Minoru Takemoto, Kazuki Kobayashi, Harukiyo Kawamura, Ryoichi Ishibashi, Koutaro Yokote, Mikiko Ohno, Po-Min Chen, Eiichiro Nishi, Koh Ono, Takeshi Kimura, Toshio Machida, Hirotaka Takizawa, Koichi Kashiwado, Hideaki Shimada, Masaaki Ito, Ken-Ichiro Goto, Katsuro Iwase, Hiromi Ashino, Akiko Taira, Emiko Arita, Masaki Takiguchi, Takaki Hiwasa

**Affiliations:** ^1^ Department of Biochemistry and Genetics, Graduate School of Medicine, Chiba University, Chiba, Japan; ^2^ Department of Anesthesia, The First Affiliated Hospital, Jinan University, Guangzhou, P. R. China; ^3^ Medical Project Division, Research Development Center, Fujikura Kasei Co., Saitama, Japan; ^4^ Department of Neurological Surgery, Graduate School of Medicine, Chiba University, Chiba, Japan; ^5^ Department of Neurological Surgery, Chiba Prefectural Sawara Hospital, Chiba, Japan; ^6^ Department of Neurosurgery, Chiba Cerebral and Cardiovascular Center, Chiba, Japan; ^7^ Department of Neurology, Chiba Rosai Hospital, Chiba, Japan; ^8^ Department of Neurology, Chibaken Saiseikai Narashino Hospital, Chiba, Japan; ^9^ Department of Internal Medicine, Chiba Aoba Municipal Hospital, Chiba, Japan; ^10^ Department of Neurology, Graduate School of Medicine, Chiba University, Chiba, Japan; ^11^ Department of Clinical Cell Biology and Medicine, Graduate School of Medicine, Chiba University, Chiba, Japan; ^12^ Department of Cardiovascular Medicine, Graduate School of Medicine, Kyoto University, Kyoto, Japan; ^13^ Department of Pharmacology, Shiga University of Medical Science, Shiga, Japan; ^14^ Port Square Kashiwado Clinic, Kashiwado Memorial Foundation, Chiba, Japan; ^15^ Department of Neurology, Kashiwado Hospital, Chiba, Japan; ^16^ Department of Surgery, School of Medicine, Toho University, Tokyo, Japan

**Keywords:** TIA, cerebral infarction, SEREX, antibody biomarker, atherosclerosis, Gerotarget

## Abstract

Transient ischemic attack (TIA) is a predictor for cerebral infarction (CI), and early diagnosis of TIA is extremely important for the prevention of CI. We set out to identify novel antibody biomarkers for TIA and CI, and detected matrix metalloproteinase 1 (MMP1), chromobox homolog 1 (CBX1), and chromobox homolog 5 (CBX5) as candidate antigens using serological identification of antigens by recombinant cDNA expression cloning (SEREX) and Western blotting to confirm the presence of serum antibodies against the antigens. Amplified luminescent proximity homogeneous assay-linked immunosorbent assay (AlphaLISA) revealed that serum antibody levels were significantly higher in patients with TIA or acute-phase CI (aCI) compared with healthy donors (*P* < 0.01). Spearman’s correlation analysis and multivariate logistic regression analysis demonstrated that levels of anti-MMP1, anti-CBX1, and anti-CBX5 antibodies were associated with age, cigarette-smoking habits, and blood pressure. Thus, serum levels of antibodies against MMP1, CBX1, and CBX5 could potentially serve as useful tools for diagnosing TIA and predicting the onset of aCI.

## INTRODUCTION

Cerebral infarction (CI), namely ischemic stroke, is the most common cerebrovascular disorder worldwide and is a major cause of fatality and disability [1-3]. The etiology of CI is not well understood because its onset results from many risk factors, such as hypertension, diabetes mellitus (DM), hyperlipidemia, atrial fibrillation, asymptomatic carotid stenosis, cigarette smoking, and alcohol consumption [4]. In addition, not all individuals exposed to similar risk factors develop CI. The CI is frequently accompanied by transient ischemic attack (TIA), which suggests that patients with TIA are at high risk of early CI [5-8]. TIA is a transient episode of neurologic dysfunction caused by focal cerebral ischemia without acute infarction. TIA has the same underlying cause as CI, that is, disruption of cerebral blood flow, and is an independent risk factor for CI [9]. Epidemiologic studies revealed that the prevalence of prior TIA ranged from 7 to 40% among patients who presented with stroke, and rates of TIA were as great as 50% among those with atherothrombotic stroke [9]. The risk of stroke after TIA was 14.6% at 3-month follow-up [10], and 5.1% at day 365 [11]. Thus, TIA is a warning for CI and is therefore also known as a mini or warning stroke. If TIA or comparable alterations are detected, CI onset can be prevented in most cases [12-15]. Therefore, attempts have been made to identify novel biomarkers to detect TIA and CI using genomics and proteomics.

It is well documented that atherosclerosis likely plays a key role in the pathogenesis of TIA and CI. Immune responses such as inflammation are associated with atherosclerosis because specific autoantibodies have been detected in the sera of patients with atherosclerosis-related diseases, such as TIA, CI, DM, cardiovascular disease (CVD), and chronic kidney disease (CKD). It is now believed that atherosclerosis results from immune responses such as inflammation and autoimmunity, which can result in damage to artery endothelial cells [16-19]. Thus, serum autoantibodies could significantly contribute to the early and sensitive diagnosis of TIA and CI.

Serological identification of antigens by recombinant cDNA expression cloning (SEREX) is an established method for identifying antigenic proteins that combines molecular cloning using phage expression libraries with serological typing [20, 21]. It is one of the most effective and convenient methods for identifying antigenic targets for various types of malignant tumors in humans on a genomic scale and has been used to find more than 1000 novel tumor antigens [22-24]. We previously performed large-scale SEREX screening and found novel tumor antigens for esophageal squamous cell carcinoma and glioma [22-35]. We also verified that the levels of spontaneous autoantibodies against SEREX antigens were useful for tumor detection. Similar expression cloning was also applied to autoimmune diseases, such as systemic lupus erythematosus, Kawasaki disease, Behçet’s disease, and multiple sclerosis [36-39]. We used SEREX for atherosclerosis-related diseases and identified antibodies against RPA2 [16] and SOSTDC1 [40] in ischemic stroke, TUBB2C [41] and adiponectin in DM [22], and ATP2B4, BMP-1 [17], DHPS [42], SH3BP5 [43], GADD34 [44], and PRCP [45] in arteriosclerosis-related diseases. In the present study, we performed SEREX screening using sera from TIA patients to identify specific and novel biomarkers of TIA with the aim of early detection of TIA as well as prediction of CI onset.

## RESULTS

### Identification of antigens recognized by serum antibodies of TIA patients

**Figure 1:**
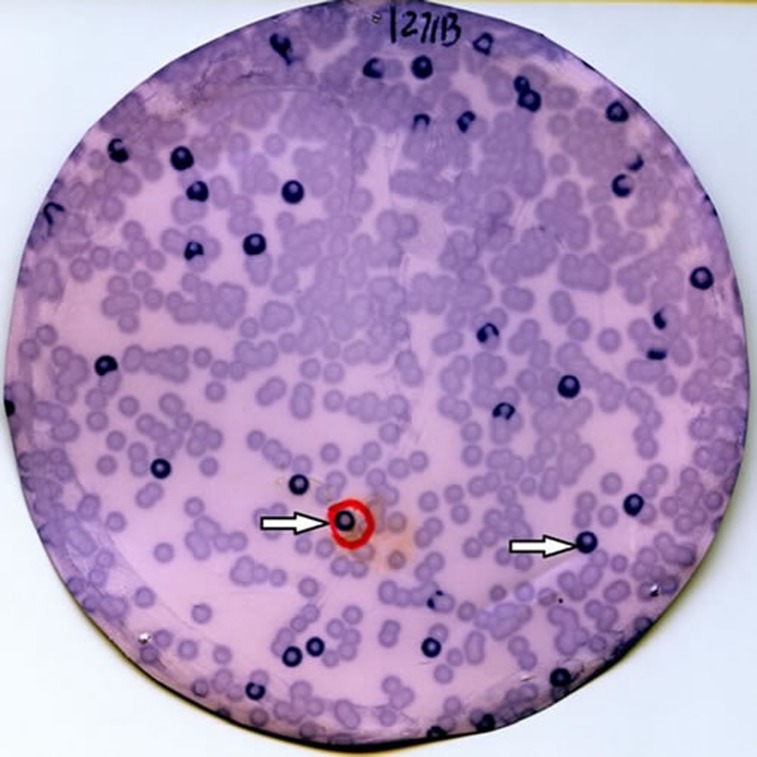
Immunoscreening of TIA antigens by SEREX Recombinant expression cloning proteins were blotted onto nitrocellulose membranes and reacted with sera from 19 TIA patients. Arrows indicate positive phage clones. Positive clones were recloned twice to obtain monoclonality.

Immunological screening was performed using the sera of 19 patients with TIA (Figure 1). Expression cloning identified three independent clones exhibiting sequence homology with matrix metalloproteinase 1 (MMP1; accession number: NM_002421), chromobox homolog 1 (CBX1; Accession number: NM_001127228), and chromobox homolog 5 (CBX5; Accession number: NM_012117). The region of MMP1 between amino acids 70 and 469 was obtained as a pBluescript II clone and recombined into a pGEX 4T-1 expression vector. Similarly, the cloned regions of CBX1 and CBX5 were amino acids 1-185 and 1-191, respectively, and both were recombined into pGEX 4T-3 vectors. Recombinant MMP1, CBX1, and CBX5 proteins were expressed in coliform bacilli as glutathione *S*-transferase (GST) fusion proteins and purified by affinity chromatography using glutathione-Sepharose.

### Presence of serum antibodies against purified proteins in patients with TIA

**Figure 2:**
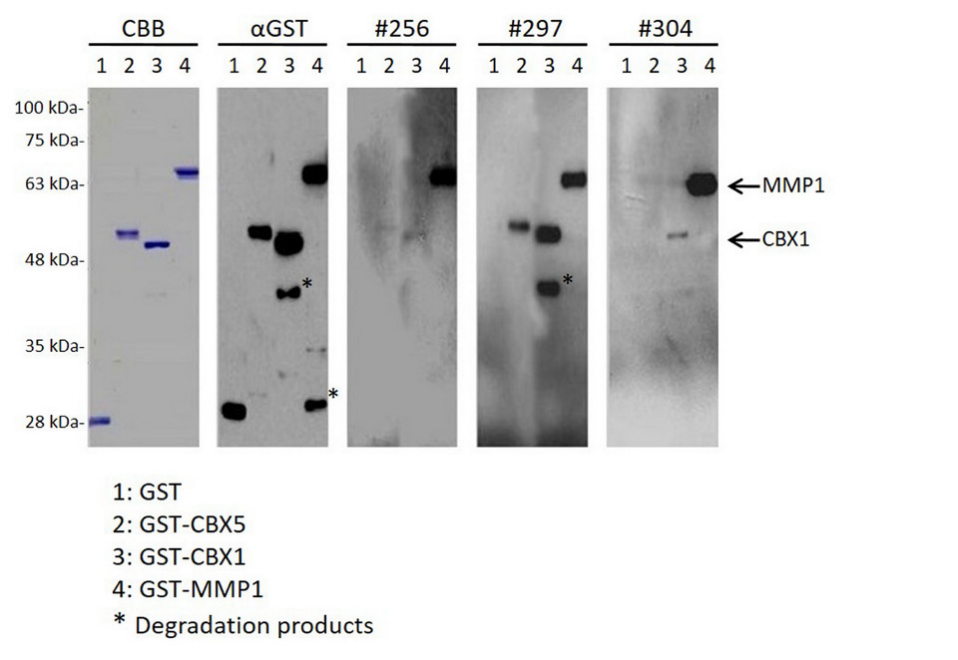
The presence of serum antibodies against MMP1, CBX1, and CBX5 antigenic proteins Representative results of Western blotting are shown, which revealed that all of the affinity-purified GST fusion antigenic proteins were detected at the expected sizes (GST-MMP1: 65 kDa; GST-CBX1: 50 kDa; and GST-CBX5: 52 kDa). GST and GST fusion proteins were electrophoresed through SDS-polyacrylamide gels followed by staining with Coomassie Brilliant Blue (CBB) or Western blotting using anti-GST (αGST) or patient sera (#256, #297, and #304). Arrows indicate specific reactions to GST-MMP1 and GST-CBX1, and the asterisk represents degradation products after electrophoresis. Molecular weights are shown to the left.

To confirm the presence of antibodies against MMP1 (MMP1-Abs), CBX1 (CBX1-Abs), and CBX5 (CBX5-Abs) in sera, Western blotting was performed using sera obtained from patients with TIA. GST-MMP1, GST-CBX1, and GST-CBX5, as well as GST proteins, were recognized using anti-GST antibodies as reactions of 65, 50, 52, and 28-kDa proteins, respectively (Figure 2). Conversely, GST-MMP1 and GST-CBX1, but not GST, reacted with the serum antibodies of patients #256, #297, and #304. GST-CBX5 was probed with sera of patients #256 and #297. Thus, most, if not all, of the reactivity of GST fusion antigen proteins with serum antibodies may be attributed to antigen proteins and not the GST domain.

### Levels of MMP1-Abs, CBX1-Abs, and CBX5-Abs are increased in patients with TIA or CI

**Figure 3:**
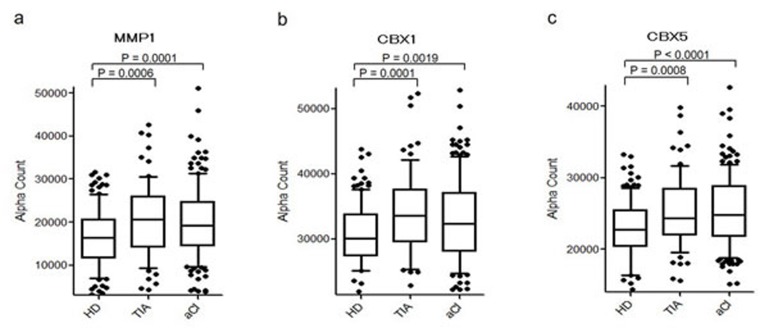
Comparison of serum MMP1-Abs, CBX1-Abs, and CBX5-Abs levels between HDs and TIA or aCI patients Antigens used were GST-MMP1 **a.**, GST-CBX1 **b.**, and GST-CBX5 **c.** Serum levels of antibodies after subtraction of the levels against control GST examined by AlphaLISA are shown using a box-whisker. The box plots display the 10th, 20th, 50th, 80th, and 90th percentiles. *P* values vs. HD specimens are shown. Table 1 shows the averages, SDs, cutoff values, total numbers, positivity numbers, positivity rates (%), and *P* values.

To quantitatively analyze the levels of MMP1-Abs, CBX1-Abs, and CBX5-Abs, we performed amplified luminescent proximity homogeneous assay-linked immunosorbent assay (AlphaLISA) using the sera of healthy donors (HDs) and patients with TIA or acute CI (aCI). AlphaLISA showed that levels of MMP1-Abs, CBX1-Abs, and CBX5-Abs were significantly higher in patients with TIA or aCI than in HDs (Figure 3a-3c), and there were no differences in the Alpha values between patients with TIA and aCI for each autoantibody (Table 1). When the cutoff value was determined as the average +2 SD of the HDs, the positivity rates for MMP1-Abs in HDs, TIA patients, and aCI patients were 2.5, 9.5, and 10.5%, respectively. The positivity rates for CBX1-Abs were 2.4, 10.4, and 11.4%, respectively, and those for CBX5-Abs were 1.6, 7.8, and 9.5%, respectively (Table 1). Thus, positivity for CBX5-Abs in TIA and aCI patients was less prominent than for MMP1-Abs or CBX1-Abs.

**Table 1:** Comparison of serum antibody levels between HDs and TIA or aCI patients examined by AlphaLISA

		MMP1	CBX1	CBX5
HD	Average	16,284	30,297	22,668
	SD	7,104	5,671	4,693
	Cutoff value	30,492	41,640	32,054
	Total number	119	123	122
	Positive number	3	3	2
	Positive rate	2.50%	2.40%	1.60%
TIA	Average	20,505	33,969	25,153
	SD	8,688	6,764	5,129
	Total number	74	77	77
	Positive number	7	8	6
	Positive rate	9.50%	**10.40%**	7.80%
	*P* (vs. HD)	**0.0006**	**0.0001**	**0.0008**
aCI	Average	19,928	32,642	25,318
	SD	8,435	6,835	5,029
	Total number	153	158	158
	Positive number	16	18	15
	Positive rate	**10.50%**	**11.40%**	9.50%
	*P* (vs. HD)	**0.0001**	**0.0019**	**8.45E-06**
	*P* (vs. TIA)	0.64	0.16	0.82

The average, SD, cutoff values (average + 2SD), total sample number, number of serum samples in which antibody levels exceeded the cutoff value, and the positivity rate (%) are presented for HDs and patients as well as *P* values of statistical comparisons between HDs and patients. The antigens used were purified GST-MMP1, GST-CBX1, and GST-CBX5 proteins. *P* values lower than 0.05 and positivity rates higher than 10% are marked in bold.

**Figure 4:**
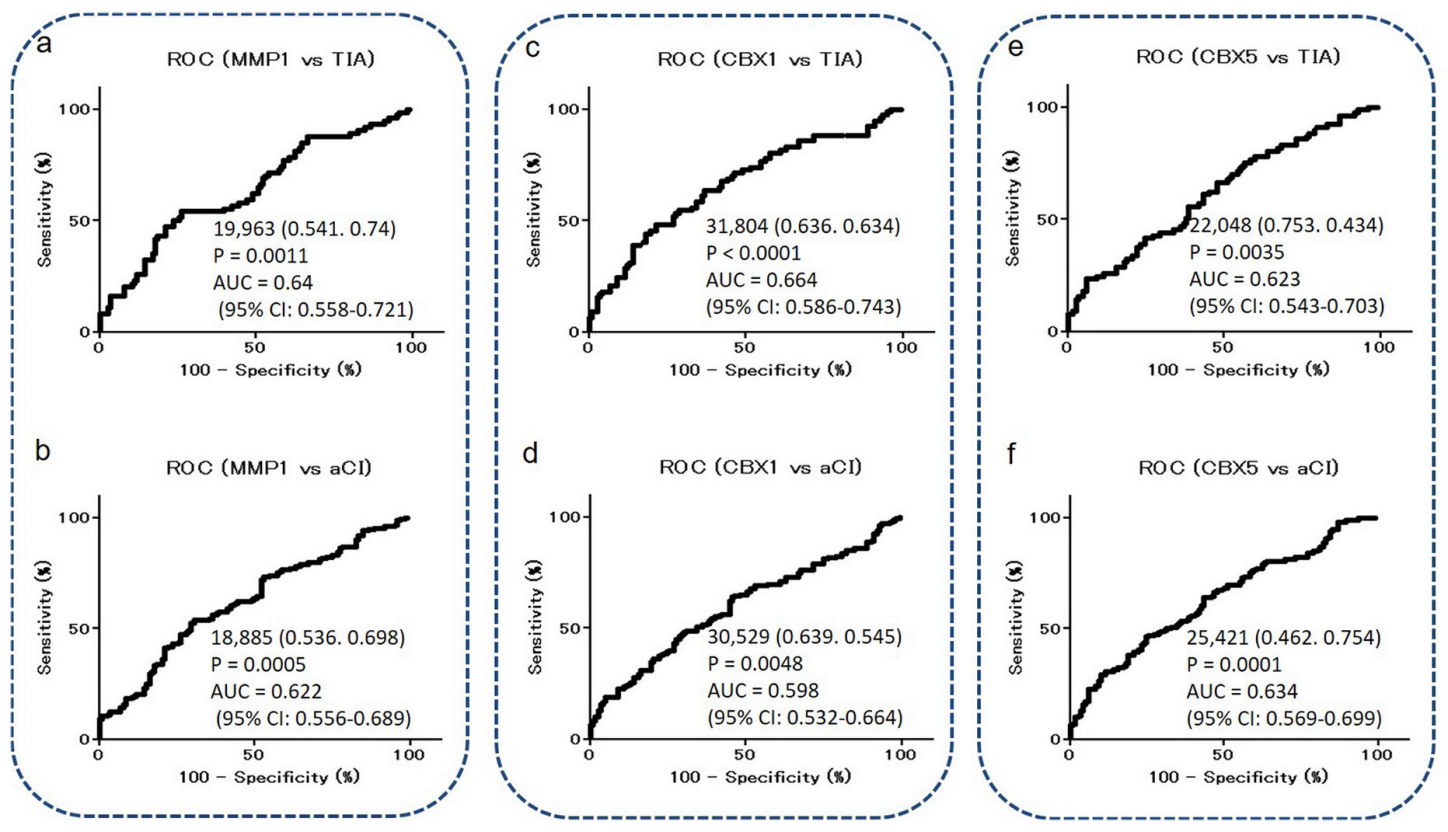
ROC analysis of MMP1-Abs, CBX1-Abs, and CBX5-Abs for the prediction of TIA or aCI Numbers in the curves indicate cutoff values for marker levels, and those in parentheses indicate sensitivity (left) and specificity (right). AUC, 95% CI, and *P* values are shown.

Receiver operating curve (ROC) analysis was performed to evaluate the ability of these markers to detect TIA and aCI. The areas under the curve (AUCs) of MMP1-Abs, CBX1-Abs, and CBX5-Abs for TIA were 0.640 [95% confidence interval (CI) = 0.558-0.721], 0.664 (95% CI = 0.586-0.743), and 0.623 (95% CI = 0.543-0.703), respectively (Figure 4a, 4c, and 4e), and those for aCI are shown in Figure 4b, 4d, and 4f. When the cutoff value for MMP1-Abs levels was determined to be 19,963, the sensitivity and specificity of the antibody levels for the diagnosis of TIA were 54.1 and 74%, respectively (Figure 4a), which were similar to the levels for the diagnosis of aCI that were 53.6 and 69.8%, respectively (Figure 4b). The largest AUC and smallest *P* value were observed for CBX1-Abs for TIA among the results shown in Figure 4.

### Levels of MMP1-Abs are elevated in acute myocardial infarction (AMI) or DM patients

**Figure 5:**
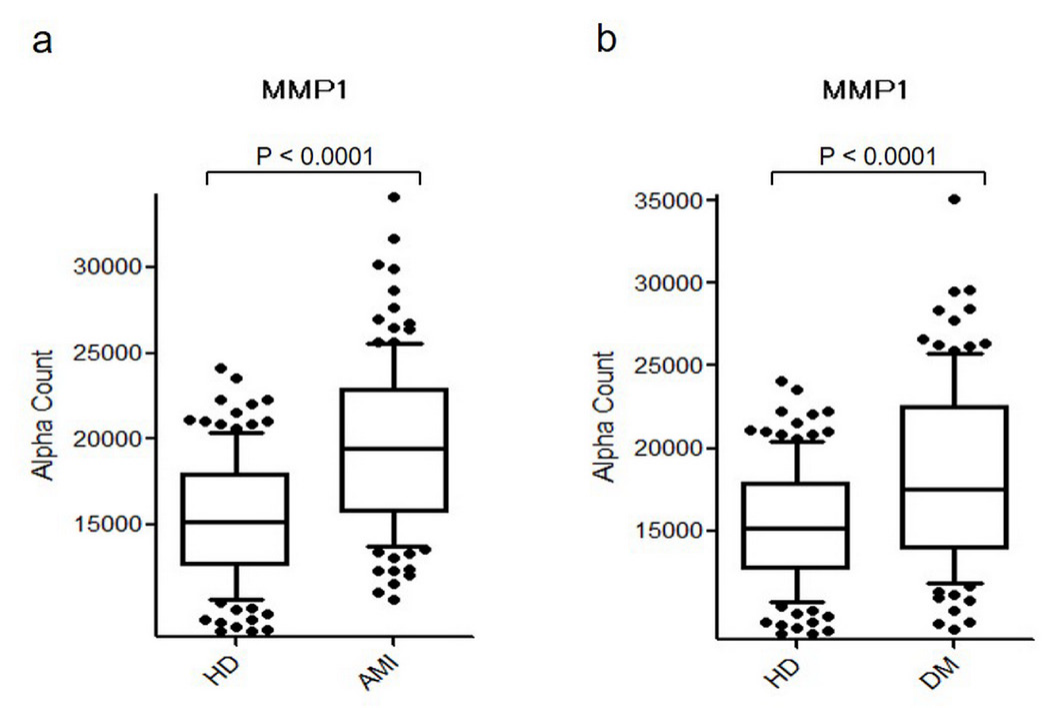
Comparison of serum MMP1-Ab levels between HDs and AMI or DM patients Serum antibody levels against MMP1 in HDs and AMI **a.** or DM **b.** patients examined by AlphaLISA are shown using a box-whisker plot. The box plots display the 10th, 20th, 50th, 80th, and 90th percentiles. Table 2 shows the averages, SDs, a cutoff value, total numbers, positivity numbers, positivity rates (%), and *P* values.

**Figure 6:**
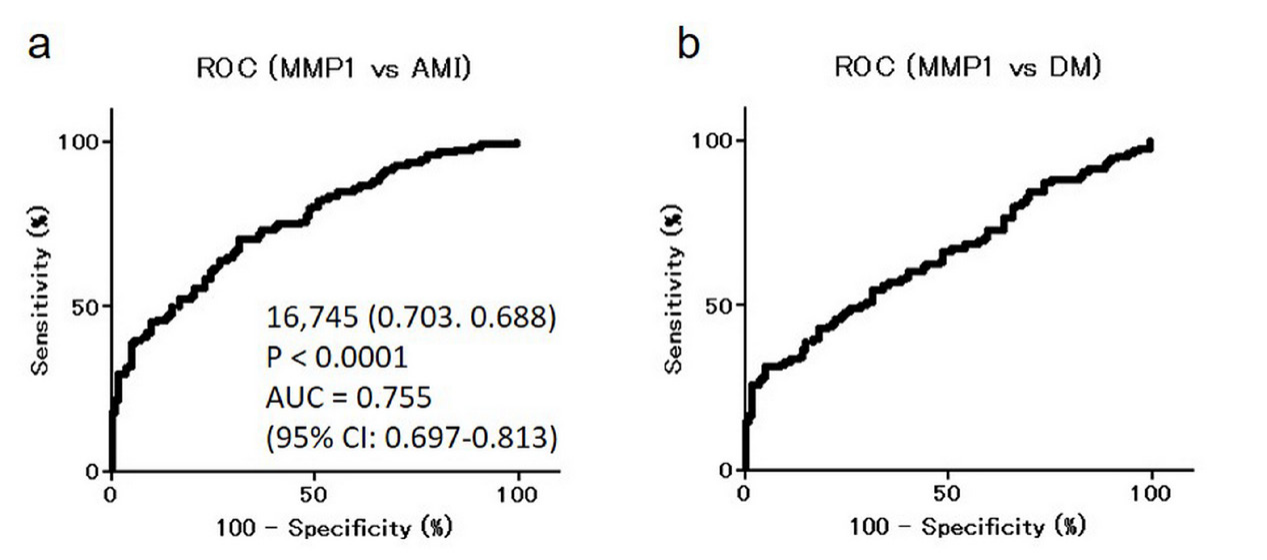
ROC analysis of MMP1-Abs levels for predicting AMI and DM ROC curves for assessing the ability of MMP1-Abs to predict AMI **a.** or DM **b.** are shown. Numbers in the figures are the same as those shown in Figure 4.

Levels of MMP1-Abs were significantly higher in AMI and DM patients compared with HDs (Figure 5a and 5b), with no difference between AMI and DM patients. The positivity rates for MMP1-Abs in HDs and AMI or DM patients were 26.6 and 23.4%, respectively (Table 2). ROC analysis revealed that the AUC for MMP1-Abs was as high as 0.755 for AMI, whereas the value for DM was similar to those for TIA and aCI (Figure 6).

**Table 2:** Comparison of MMP1 antibody levels between HDs and AMI or DM patients examined by AlphaLISA

		MMP1
HD	Average	15,338
	SD	3,655
	Cutoff value	22,649
	Total number	128
	Positive number	2
	Positive rate	1.60%
AMI	Average	19,578
	SD	4,766
	Total number	128
	Positive number	34
	Positive rate	**26.60%**
	*P* (vs. HD)	**5.95E-14**
DM	Average	18,306
	SD	5,897
	Total number	128
	Positive number	30
	Positive rate	**23.40%**
	*P* (vs. HD)	**2.49E-06**
	*P* (vs. AMI)	0.059

The antigens used were purified GST-MMP1 proteins. See Table 1 for further details.

### Levels of CBX5-Abs are associated with DM

**Figure 7:**
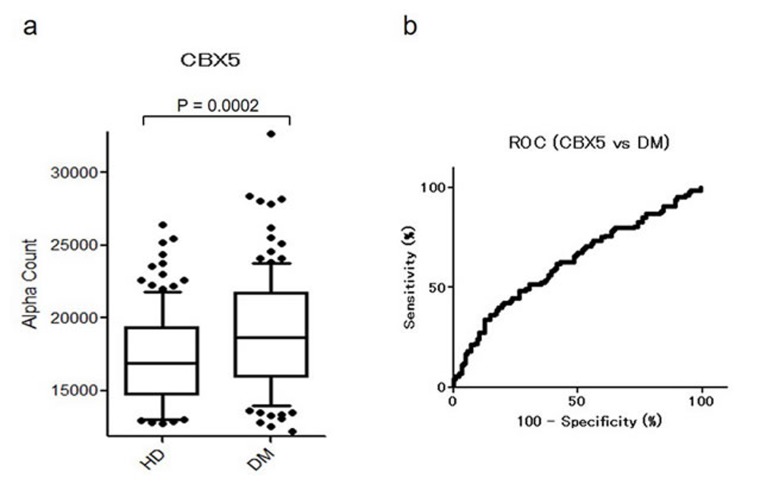
Comparison of serum CBX5-Abs levels between HDs and DM patients Serum antibody levels against CBX5 in HDs and DM patients examined by AlphaLISA are shown by a box-whisker plot **a.** The box plots display the 10th, 20th, 50th, 80th, and 90th percentiles. Table 3 shows averages, SDs, one cutoff value, total numbers, positivity numbers, positivity rates (%), and *P* values. The results were also evaluated by ROC analysis **b.**

We also measured levels of CBX1-Abs and CBX5-Abs in serum samples of HDs and patients with AMI or DM. There were no apparent differences in CBX1 between HDs and AMI or DM patients (data not shown). There were also no differences in CBX5-Abs between HDs and AMI patients, but there was a significant association with DM (Table 3 and Figure 7). The AUC and *P* value for CBX5-Abs were 0.628 and 0.0004, respectively (Figure 7b), for DM, which was comparable to those for TIA and aCI.

**Table 3:** Comparison of CBX5 antibody levels between HDs and DM patients examined by AlphaLISA

		CBX5
HD	Average	17,132
	SD	3,333
	Cutoff value	23,799
	Total number	128
	Positive number	4
	Positive rate	3.10%
DM	Average	18,909
	SD	4,083
	Total number	128
	Positive number	12
	Positive rate	9.40%
	*P* (vs. HD)	**0.0002**

The antigens used were purified GST-CBX5 proteins. See Table 1 for further details.

### Correlation analysis between serum antibody levels and risk factors for CI

We performed Spearman’s correlation analysis and multivariate logistic regression analysis between antibody marker levels and data on the study individuals, including gender, age, height, weight, body mass index, blood pressure, smoking habits, alcohol drinking habits, alcohol consumption, and working strength. Blood biochemistry was also assessed as previously described [17, 40-43], including white blood cells, red blood cells, platelets, total cholesterol (TC), high-density lipoprotein-cholesterol (HDL-c), low-density lipoprotein-cholesterol (LDL-c), triglycerides (TG), total protein, albumin, total bilirubin, aspartate aminotransferase, alanine aminotransferase, γ-glutamyltranspeptidase, alkaline phosphatase, lactate dehydrogenase, uric acid, C-reactive protein, and glycated hemoglobin. A total of 357 specimens were examined, including 123 specimens obtained from HDs, 77 from TIA patients, and 157 from aCI patients. Both analyses suggested that levels of MMP1-Abs were correlated with age and cigarette smoking habits (duration), but were inversely correlated with working strength. Levels of CBX1 were inversely correlated with TC, whereas those of CBX5 were associated with blood pressure (Table 4). None of the other patient data were significantly correlated with levels of MMP1-Abs, CBX5-Abs, and CBX5-Abs.

**Table 4:** Correlation analysis between serum antibody marker levels and the indices in HDs, TIA and aCI patients.

	MMP1				CBX1				CBX5			
	Spearman		Multivariate		Spearman		Multivariate		Spearman		Multivariate	
	r value	*P value*	r value	*P value*	r value	*P value*	r value	*P value*	r value	*P value*	r value	*P value*
Gender	0.031	0.5712	0.069	0.2207	0.063	0.2356	-0.012	0.8291	-0.054	0.3055	0.111	**0.0440**
Age	0.191	**0.0004**	-0.146	**0.0096**	0.253	**< 0.0001**	0.056	0.3073	0.189	**0.0003**	0.062	0.2658
Height	-0.072	0.1887	0.010	0.8622	-0.106	**0.0474**	-0.114	**0.0385**	0.012	0.8243	-0.074	0.1803
Weight	-0.023	0.6786	-0.013	0.8206	-0.034	0.5212	0.100	0.0704	0.054	0.3149	0.105	0.0574
BMI	0.009	0.8676	0.006	0.9086	0.032	0.5468	-0.087	0.1168	0.047	0.3829	-0.100	0.0692
Blood pressure	0.117	**0.0482**	0.006	0.9212	0.086	0.1399	-0.114	**0.0391**	0.158	**0.0064**	0.173	**0.0016**
Smoking	0.163	**0.0025**	-0.076	0.1759	0.117	**0.0278**	-0.024	0.6581	0.219	**< 0.0001**	0.103	0.0628
Smoking period	0.237	**< 0.0001**	0.136	**0.0156**	0.187	**0.0005**	-0.006	0.9174	0.264	**< 0.0001**	0.058	0.2902
Alcohol	-0.033	0.5469	0.102	0.0709	-0.100	0.0602	0.053	0.3354	-0.026	0.6209	-0.026	0.6365
Alcohol frequency	-0.002	0.9677	-0.123	**0.0282**	-0.066	0.2204	-0.063	0.2543	0.059	0.2683	0.020	0.7142
Working	-0.187	**0.0007**	-0.112	**0.0460**	-0.200	**0.0002**	-0.013	0.8076	-0.130	**0.0174**	-0.037	0.5033
WBC	0.128	**0.0189**	0.031	0.5887	0.007	0.9028	0.020	0.7177	0.132	**0.0141**	-0.086	0.1182
RBC	-0.005	0.9287	0.007	0.9005	-0.016	0.7713	-0.058	0.2969	0.040	0.4629	-0.034	0.5357
PLT	-0.133	**0.0158**	-0.069	0.2209	-0.125	**0.0212**	-0.033	0.5535	-0.142	**0.0089**	-0.066	0.2325
TC	-0.141	**0.0180**	0.040	0.4756	-0.133	**0.0226**	-0.174	**0.0015**	-0.107	0.0668	-0.065	0.2429
HDL-c	-0.081	0.2093	-0.030	0.5976	-0.111	0.0795	0.031	0.5729	-0.109	0.0847	0.013	0.8109
LDL-c	-0.129	**0.0429**	-0.076	0.1767	-0.069	0.2748	0.177	**0.0012**	-0.063	0.3149	0.021	0.7043
TG	0.049	0.4354	0.042	0.4590	0.041	0.5019	0.009	0.8674	0.123	**0.0456**	0.091	0.0999
Total protein	0.039	0.4854	0.024	0.6738	0.028	0.6071	0.136	**0.0133**	-0.012	0.8340	-0.081	0.1448
Albumin	0.003	0.9601	0.001	0.9879	-0.044	0.4319	-0.080	0.1485	-0.013	0.8229	0.135	**0.0142**
tBil	-0.035	0.5350	-0.025	0.6592	-0.007	0.8944	-0.009	0.8767	-0.012	0.8325	0.009	0.8643
AST	0.051	0.3490	0.106	0.0602	0.015	0.7746	0.051	0.3548	0.075	0.1649	0.098	0.0747
ALT	-0.044	0.4192	-0.113	**0.0452**	-0.096	0.0743	-0.138	**0.0125**	0.001	0.9781	-0.094	0.0873
γ-GTP	0.047	0.4177	-0.025	0.6538	0.028	0.6177	0.090	0.1014	0.139	**0.0134**	-0.010	0.8506
ALP	0.110	0.0785	0.012	0.8255	0.096	0.1175	0.026	0.6382	0.108	0.0796	0.011	0.8398
LDH	0.134	**0.0181**	0.019	0.7343	0.093	0.0970	-0.052	0.3434	0.141	**0.0117**	0.100	0.0706
UA	0.019	0.7571	0.098	0.0815	0.072	0.2247	0.176	**0.0013**	0.104	0.0803	-0.046	0.4086
CRP	0.114	0.0699	-0.127	**0.0236**	0.066	0.2821	-0.117	**0.0343**	0.154	**0.0116**	0.028	0.6133
HbA1C	0.067	0.2951	-0.027	0.6277	0.097	0.1193	0.016	0.7755	0.066	0.2884	0.073	0.1855

The data on study individuals were obtained from HD subjects in Chiba Prefectural Sawara Hospital and Port Square Kashiwado Clinic and TIA or aCI patients in Chiba Prefectural Sawara Hospital, Chiba Rosai Hospital, and Chiba Aoba Municipal Hospital. Correlation coefficients (r) and *P* values were calculated via Spearman’s correlation analysis and multivariate logistic regression analysis. *P* values less than 0.05 are marked in bold.

## DISCUSSION

Atherosclerosis is one of the major causes of TIA and CI, and current studies suggest that both innate and adaptive types of immunity are involved in the development of atherosclerotic lesions [18, 19]. For example, autoantibodies, such as those against RPA2, ATP2B4, and TUBB2C have been identified as atherosclerosis and/or CI markers [16, 17, 41]. The onset of CI may induce the spread of various antigens; however, antibodies against these antigens are not detectable until at least 2 weeks after disease onset. Therefore, the antibodies that were specifically detected in patients immediately after onset of CI were probably present prior to the disease. Consequently, these antibodies have been suggested to be predictive markers for the onset of CI [16, 17, 40, 41]. If this is the case, the levels of these autoantibodies may increase at the stage of TIA, which is frequently a warning for CI [5-9]. Thus, to identify antibody markers useful for the diagnosis of TIA, as well as prediction of CI onset, we performed SEREX screening using serum samples from TIA patients.

In this study, three SEREX antigens—MMP1, CBX1, and CBX5—were identified, and the presence of their autoantibodies was confirmed by Western blotting (Figure 2). AlphaLISA enabled us to quantitate the precise levels of these serum antibodies, and the results illustrated that the antibody levels of anti-MMP1, anti-CBX1, and anti-CBX5 were significantly elevated not only in aCI but also in TIA patients compared with HDs (Figure 3). In particular, increased levels of these antibodies as early as the TIA stage suggest that they are predictive markers for aCI.

MMPs, which are capable of degrading and remodeling all major components of the extracellular matrix (ECM), comprise a family of structurally related zinc-dependent endopeptidases excreted by a number of cell types *in vivo*. MMP1 (also known as collagenase) is implicated in the mechanisms underlying membrane rupture [1, 46, 47]. Recently, considerable attention has been paid to the important role of MMPs play in cartilage degeneration, which starts from ECM metabolism imbalance involving proteoglycan degradation and collagen damage [48, 49]. Degradation of the ECM is also involved in the incipient stage of atherosclerosis and occurs over the course of the progressive disease [50, 51]. Therefore, MMP1 may have a subtle regulatory effect on the pathogenesis of atherosclerosis. Consistently, MMP1-Abs levels were associated with aCI, TIA, AMI, and DM, all of which are related to atherosclerosis (Figures 3-6). These results suggest that MMP1-Abs could be a common marker of atherosclerosis-related diseases.

CBX1 and CBX5 are members of the heterochromatin protein 1 (HP1) family, which is evolutionarily conserved [52]. The HP1 family of non-histone chromosomal proteins is involved in the establishment and maintenance of higher-order chromatin structures [52-54]. The most recent accomplishments indicated that HPs are closely related to the development of major vascular injuries, such as atherosclerosis and hypertension because genes that are silenced in differentiated vascular smooth muscle cells acquire modifications associated with a closed chromatin state (i.e., heterochromatin) [55]. Therefore, HP1 family members may play a regulatory role in the development of atherosclerosis. Consistently, levels of CBX1-Abs and CBX5-Abs were elevated in TIA and CI patients (Figures 3 and 4). In addition, there was an increase in the small aggregation of heterochromatin and a decrease in the level of interchromatin granules in the nuclei of fibroblasts from patients with atypical Werner syndrome (AWS), which is usually accompanied by disorders with multiple features resembling accelerated aging, mainly including vascular diseases such as aortosclerosis and DM [56, 57]. This suggests that abnormal nuclear morphology and chromatin disorganization may be associated with the pathogenesis of AWS/DM, and studies into the functional role of HP1 family members, particularly CBX5, which has diverse roles in the nucleus, including heterochromatin packaging and euchromatic gene regulation [52, 53], may be important. Thus, the expression of CBX5 appears to be associated with DM, which may explain our findings that levels of CBX5-Abs were higher in patients with DM as well as TIA or aCI patients than in HDs (Figures 3, 4, and 7).

The significantly higher levels of MMP1-Abs and CBX5-Abs in patients with DM compared with HDs (Figures 5-7) raised the possibility that the association of these markers with TIA and aCI may have resulted from their association with DM. We next examined whether the positivity rates were different between patients with and without DM using a chi-squared test. The results uncovered no significant differences in the levels of MMP1-Abs, CBX1-Abs, and CBX5-Abs between TIA patients with and without DM (Supplementary Table 1). Similarly, there was no apparent difference in these levels between aCI patients with and without DM. Consequently, these markers may reflect a CI-prone state rather than resulting in diseases caused by DM.

It is well known that there are many factors that affect the occurrence and development of atherosclerosis, including age, smoking habits, DM, and hypertension [4]. Spearman’s correlation analysis and multivariate logistic regression analysis suggested that levels of MMP1-Abs, CBX1-Abs, and CBX5-Abs reflected different aspects of patients, that is, MMP1-Abs reflected age and smoking duration, CBX1-Abs reflected total cholesterol, and CBX5-Abs reflected blood pressure/hypertension (Table 4). Levels of MMP1-Abs showed an inverse correlation with working strength, which was inversely related to age. Thus, MMP1-Abs may discriminate aging-associated diseases, which is consistent with the results showing elevated levels of MMP1-Abs in all aCI, TIA, AMI, and DM groups (Figures 3-6). Smoking habits may accelerate aging. On the other hand, CBX5-Abs may discriminate aCI and TIA induced by DM and/or hypertension, and could reflect the abnormal state of cholesterol. Consequently, each antibody marker can detect a certain type of aCI and TIA, possibly reflecting the causal abnormality, and therefore, examination using combinations of multiple markers may improve the sensitivity.

We identified three antibody markers for TIA by SEREX screening. AlphaLISA using recombinant antigen proteins demonstrated that the levels of antibodies against MMP1, CBX1, and CBX5 were higher in patients with TIA or aCI than in HDs, suggesting that these antibody markers are valuable for the diagnosis of TIA and aCI. Antibody markers may be more sensitive than antigen markers because repeated exposure of a small amount of an antigen to immune cells results in the production of a large amount of the antibody. Therefore, increasing numbers of antibody markers, such as those against oxidized LDL for atherosclerosis [58], p53 antibodies for cancer [59], and glutamic acid decarboxylase antibodies for type-1 DM [60], have been developed for practical use. However, antibody markers have some limitations. Autoantibodies are frequently observed in patients with cancer and autoimmune diseases. Further studies, including those of cancer and autoimmune diseases, are necessary for practical diagnostic use.

In conclusion, the association of serum levels of antibodies against MMP1, CBX1, and CBX5 could potentially represent useful tools for the diagnosis of TIA and potential prediction of the onset of aCI.

## MATERIALS AND METHODS

### Ethics statement

This study was approved by the Local Ethical Review Board of the Graduate School of Medicine, Chiba University, as well as those of co-operating hospitals and was performed in accordance with the principles of the Declaration of Helsinki. Recombinant DNA studies were performed with the official permission of the Graduate School of Medicine, Chiba University, and conducted in conformity with the rules of the Japanese government. Written informed consent was obtained from all participants.

### Participants

To identify biomarkers for TIA and CI, serum was collected from HDs and patients diagnosed with TIA, aCI, AMI, or DM. HDs were used as normal controls and were selected from patients who underwent a medical checkup; all HDs were without a history of TIA, aCI, AMI, and DM. The inclusion criteria were a history of TIA, aCI, AMI, or DM. Serum samples from patients with TIA, aCI, or AMI were collected within 2 weeks of disease onset. Patients with DM having undergone anti-diabetic therapy or having a history of diabetes were selected. Subjects with an autoimmune disease were excluded from this study.

For immunological screening by SEREX, 19 patients with TIA were randomly selected (three women and 16 men; mean age, 68.26 ± 10.23 years). To detect serum antibody markers for TIA and CI by AlphaLISA, three independent cohorts (HD, TIA, and aCI groups) were studied (see Table 5 for baseline characteristics of participants). Because the primary mechanism of cerebrovascular diseases, including TIA and CI, is atherosclerosis, which is closely related to AMI and DM, we also compared antibody levels between 128 HDs and 128 patients with AMI or DM. The average age of HDs, patients with AMI, and patients with DM were 58.29 ± 5.63, 58.2 ± 8.5, and 58.37 ± 9.11 years, respectively.

**Table 5:** Baseline characteristics of participants in HD, TIA and aCI groups

	HD	TIA	aCI
	(Total of 123)	(Total of 77)	(Total of 158)
Male gender	85 (69.1)	48 (62.3)	119 (75.3)
Age	51.85 ± 8.75	69.6 ± 11.74	57.67 ± 7.61
Height	165.86 ± 8.72	158.79 ± 10.41	163.79 ± 8.45
Weight	65.67± 13.06	59.68 ± 11.71	64.81 ± 12.46
Body mass index	23.77 ± 3.98	23.67 ± 3.46	24.1 ± 4
Smoking	62 (50.4)	42 (54.5)	105 (66.5)
Hypertension	15 (12.2)	48 (62.3)	90 (57)
DM	0	18 (23.4)	42 (26.6)

The HDs were from Chiba Prefectural Sawara Hospital and Port Square Kashiwado Clinic. The TIA or aCI patients were from Chiba Prefectural Sawara Hospital, Chiba Rosai Hospital, and Chiba Aoba Municipal Hospital. Classical risk factors for atherosclerosis, including gender, age, body mass index, smoking, incidence of hypertension and diabetes, were evaluated from clinical records. Participants were considered as smoking if they currently smoked or had a history of smoking. Hypertension was defined as a history of blood pressure above 140 mmHg in systolic or 90 mmHg in diastolic pressure or the use of antihypertensive agents. DM was defined as having undergone antidiabetic therapy or having a history of diabetes. Data expresses as the average ± standard deviation for numerical data and n (%) for categorical data.

### Serum samples

Serum samples, with or without ethylenediaminetetraacetic acid (EDTA), were collected from each participant upon admission. The serum samples of patients with TIA or CI in the acute phase were obtained from Chiba Prefectural Sawara Hospital, Chiba Rosai Hospital, and Chiba Aoba Municipal Hospital. Serum samples from patients with AMI were obtained from Kyoto University Hospital, and those of patients with DM were obtained from Chiba University Hospital. Serum samples of HDs were obtained from Chiba Prefectural Sawara Hospital and Port Square Kashiwado Clinic. HDs were selected from among subjects who exhibited no abnormalities on cranial magnetic resonance imaging. Each serum sample was centrifuged at 3000×*g* for 10 min, and the supernatant was stored at −80°C until used. Repeated thawing and freezing of samples was avoided.

### Immunoscreening of TIA antigens

Immunoscreening was performed using a modification of previously published methods [16, 17, 22-35, 39, 42, 43]. We used a commercially available human aortic endothelial cell cDNA expression library (Uni-ZAP XR Premade Library, Stratagene, La Jolla, CA) to screen for clones that were immunoreactive against serum IgG from patients with TIA. *Escherichia coli* XL1-Blue MRF′ was infected with Uni-ZAP XR phages, and the expression of resident cDNA clones was induced by blotting the infected bacteria onto nitrocellulose membranes (NitroBind, Osmonics Inc., Minnetonka, MN) pretreated with 10 mM isopropyl-β-D-thiogalactoside (IPTG, Wako Pure Chemicals, Osaka, Japan) for 30 min. Membranes adhered with bacterial proteins were rinsed three times using TBS-T [20 mM Tris-HCl (pH 7.5), 0.15 M NaCl, and 0.05% Tween 20], and non-specific binding was blocked by incubating with 1% protease-free bovine serum albumin (Nacalai Tesque, Inc., Kyoto, Japan) in TBS-T for 1 h. Membranes were then exposed to sera (diluted 1:2000) from 19 patients with TIA for 1 h. After washing with TBS-T three times again, the membranes were incubated with 1:5000-diluted alkaline phosphatase-conjugated goat anti-human IgG (Jackson ImmunoResearch Laboratories, West Grove, PA) for 1 h. Positive reactions were visualized by incubation in color development solution [100 mM Tris-HCl (pH 9.5), 100 mM NaCl, and 5 mM MgCl_2_] containing 0.15 mg/mL 5-bromo-4-chloro-3-indolyl phosphate (Wako Pure Chemicals) and 0.3 mg/mL nitroblue tetrazolium (Wako Pure Chemicals). Positive clones were recloned twice to obtain monoclonality, as previously described.

### Sequence analysis of identified antigens

Monoclonal phage cDNA clones were converted to pBluescript phagemids by single-clone excision using the ExAssist helper phage (Stratagene). The pBluescript plasmids containing inserted cDNAs were obtained from the *E. coli* SOLR strains transformed by the phagemids. The cDNA inserts were sequenced and analyzed for homology with known genes or proteins from the RefSeq Database at the National Center for Biotechnology Information using Basic Local Alignment Search Tool (http://www.ncbi.nlm.nih.gov/Blast.cgi/).

### Construction of expression vectors

Expression plasmids of GST fusion proteins were constructed by recombining the cDNA sequences into pGEX-4T vectors (GE Healthcare Life Sciences, Pittsburgh, PA) as previously described [16, 17, 23-26, 28-32, 34, 39, 42, 43]. The pBluescript plasmids containing cDNA inserts were digested with *Eco*RI and *Xho*I and separated via agarose gel electrophoresis. The inserted cDNA fragments were isolated using GenElute Minus EtBr Spin Columns (Sigma-Aldrich, St. Louis, MO). Using Ligation Convenience Kits (Nippon Gene, Toyama, Japan), the inserts were properly ligated in frame to *Eco*RI- and *Xho*I-digested pGEX-4T-1 or pGEX-4T-3 linearized vectors, which express the recombinant GST-tagged proteins. The ligation mixtures were used to transform ECOS competent *E. coli* BL-21 cells (Nippon Gene), and appropriate recombinants were confirmed by DNA sequence analysis as well as protein expression. To confirm successful recombination, expression of GST fusion proteins was induced by treating the transformed bacterial clones with 0.1 mM IPTG for 2.5 h, and expressed proteins were electrophoresed through 11% sodium dodecyl sulfate (SDS)-polyacrylamide gels.

### Purification of recombinant candidate proteins

Transformed *E. coli* BL-21 cells containing pGEX-4T clones were cultured in 200 mL of Luria broth and treated with 1 mM IPTG for 3 h. The IPTG-treated cells were then harvested, washed with phosphate-buffered saline (PBS), and lysed by sonication in BugBuster Master Mix (Novagen, San Diego, CA). Subsequently, cell lysates were centrifuged at 13,000×*g* for 10 min at 4°C. The GST fusion recombinant proteins recovered in the supernatant fraction were directly affinity purified by glutathione-Sepharose column chromatography (GE Healthcare Life Sciences), according to the manufacturer’s instructions, and the purified proteins were concentrated using Amicon Ultra-15 Centrifugal Filter Devices (Merck Millipore, Darmstadt, Germany). The precipitates containing recombinant proteins were dissolved in 8 M urea in TED buffer [50 mM Tris-HCl (pH 8.0), 1 mM EDTA, 1 mM dithiothreitol], followed by dialysis stepwise against 4 and 2 M urea in TED buffer for 1 h each. The samples were then dialyzed against TED buffer for more than 12 h and centrifuged at 10,000×*g* for 30 min at 4°C. The recombinant proteins recovered in the supernatant were purified using glutathione-Sepharose as previously described [16, 17, 25, 28, 42, 43].

### Western blotting analysis

GST and GST fusion proteins (0.3 μg) were separated by SDS-polyacrylamide gel electrophoresis and electrically transferred onto nitrocellulose membranes. The membranes were blocked using blocking solution [0.5% skim milk powder in a buffer comprising 20 mM Tris-HCl (pH 7.6), 137 mM NaCl, and 0.1% Tween 20], and the blotted proteins were probed with specific primary antibodies, that is, antibodies against GST (goat) (Rockland, Gilbertsville, PA) or 1:5000-diluted sera from TIA patients (#256, #297, and #304), for which candidate SEREX antigenic proteins had been identified. After incubation with horseradish peroxidase (HRP)-conjugated secondary antibody (donkey anti-goat or anti-human IgG, Santa Cruz Biotechnology, CA), immunoreactivity was detected with Immobilon Western HRP Substrate (Merck Millipore) as previously described [16, 17, 34, 42, 43].

### AlphaLISA of antibody biomarkers

For the quantitative measurement of serum antibodies against the purified proteins, AlphaLISA was performed using 384-well microtiter plates (white opaque OptiPlate, Perkin Elmer) containing 2.5 μL of 1:100-diluted sera and 2.5 μL of GST or GST fusion proteins (10 μg/mL) in AlphaLISA buffer (25 mM HEPES, pH 7.4, 0.1% casein, 0.5% Triton X-100, 1 mg/mL Dextran-500, and 0.05% Proclin-300). The reaction mixture was incubated for 6-8 h at room temperature. Subsequently, anti-human IgG-conjugated acceptor beads (2.5 μL, 40 μg/mL) and glutathione-conjugated donor beads (2.5 μL, 40 μg/mL) were added, and the mixture incubated for 7-14 days at room temperature in the dark. The chemical emission was read on an EnSpire Alpha microplate reader (Perkin Elmer), as previously described [17, 39, 40-45]. Specific reactions were calculated by subtracting the Alpha values (Alpha counts) of the GST control from those of GST fusion proteins.

### Statistical analyses

The differences in the Alpha values between two groups were compared using Student’s *t*-test and Mann-Whitney *U* test. The correlation between Alpha values and data on study individuals was determined using Spearman’s correlation analysis and multivariate logistic regression analysis. The predictive values of markers for diseases were assessed by ROC analysis, and the cutoff values were set to maximize the sum of sensitivity and specificity. Comparisons of serum levels of antibody in patients with and without DM were performed using the chi-squared test. All tests were two-tailed, and *P* < 0.05 was considered statistically significant. All statistical analyses were performed using GraphPad Prism 5 (GraphPad Software, La Jolla, CA).

## SUPPLEMENTARY MATERIALS TABLE


