# Supplementary material for: Association of serum levels of antibodies against MMP1, CBX1, and CBX5 with transient ischemic attack and cerebral infarction
Source: Oncotarget. 2017 Dec 31;9(5):5600–13. doi: 10.18632/oncotarget.23789 (PMC5814161; doi:10.18632/oncotarget.23789)
Supplement: Supplementary file 1 [file oncotarget-09-5600-s001.pdf]

Association of serum levels of antibodies against MMP1, CBX1, and CBX5 with transient ischemic attack and cerebral infarction

SUPPLEMENTARY MATERIALS

Supplementary Table 1: Comparison of serum antibody levels in patients with or withoug DM using chi-square test.

TIA & aCI (Total of 197)		MMP1		CBX1		CBX5	
		High value	Low value	High value	Low value	High value	Low value
TIA (Total of 71)	With DM	12 (67%)	6 (33%)	13 (72%)	5 (28%)	14 (78%)	4 (22%)
	Without DM	27 (54%)	23 (46%)	34 (64%)	19 (36%)	39 (74%)	14 (26%)
	P value	0.3515		0.5317		0.7239	
aCI (Total of 126)	With DM	24 (59%)	17 (41%)	26 (62%)	16 (38%)	18 (43%)	24 (57%)
	Without DM	44 (54%)	37 (46%)	54 (64%)	30 (36%)	42 (50%)	42 (50%)
	P value	0.6579		0.7936		0.4492	

The TIA or aCI patients were from Chiba Prefectural Sawara Hospital and Chiba Rosai Hospital. As shown in below: total patient number, number of high value (number of positive patients of which the antibody levels were higher than the cutoff value), number of low value (number of negative patients of which the antibody levels were lower than the cutoff value), and the positive rate (%) of patients; and P values of statistical comparison between patients with DM and patients without DM using Chi-square test. The cut-off values were setting at the value that maximize the sums of the sensitivity and specificity by ROC analysis. In Figure 4, cut-off values are shown.
